# Evaluation of the power and type I error of recently proposed family-based tests of association for rare variants

**DOI:** 10.1186/1753-6561-8-S1-S36

**Published:** 2014-06-17

**Authors:** Allison Hainline, Carolina Alvarez, Alexander Luedtke, Brian Greco, Andrew Beck, Nathan L Tintle

**Affiliations:** 1 Department of Statistics, Baylor University, 1311 S 5th St., Waco, TX 76798, USA; 2 Department of Biostatistics, Florida International University, 11200 SW 8th St., Miami, FL 33199, USA; 3 Divison of Biostatistics, University of California, Berkeley, 101 Sproul Hall, Berkeley, CA 94720, USA; 4 Department of Mathematics and Statistics, Grinnell College, 733 Broad St., Grinnell, IA 50112, USA; 5 Department of Mathematics, Loyola University Chicago, 1032 W. Sheridan Rd, Chicago, IL 60660, USA; 6 Department of Mathematics, Statistics and Computer Science, 498 4th Ave. NE, Dordt College, Sioux Center, IA 51250, USA

## Abstract

Until very recently, few methods existed to analyze rare-variant association with binary phenotypes in complex pedigrees. We consider a set of recently proposed methods applied to the simulated and real hypertension phenotype as part of the Genetic Analysis Workshop 18. Minimal power of the methods is observed for genes containing variants with weak effects on the phenotype. Application of the methods to the real hypertension phenotype yielded no genes meeting a strict Bonferroni cutoff of significance. Some prior literature connects 3 of the 5 most associated genes (*p *<1 × 10^−4^) to hypertension or related phenotypes. Further methodological development is needed to extend these methods to handle covariates, and to explore more powerful test alternatives.

## Background

The advent of next-generation sequencing technology has allowed researchers to actively consider the impact of rare genetic variation on common disease. However, novel statistical methodologies have been needed to leverage the limited evidence provided by any single-nucleotide rare genetic variant (SNV). Two major classes of tests, collapsing and variance components, have been proposed for testing case-control association (see Ref. [1] for an overarching framework that classifies and describes the general behavior of the tests). Virtually all of the recently proposed methods use the gene as the unit of analysis with the goal of aggregating independent genotype-phenotype association signals across the set of SNVs in the gene to boost statistical power versus the limited potential power from individually testing millions of SNVs, many of which may be extremely rare (eg, singletons). This SNV-set testing approach is not only statistically reasonable, it is also biologically plausible, because it may be that separate, independent variations within the protein encoding region of a gene could alter protein structure and, ultimately, explain phenotypic diversity.

Practically, however, many of these methods have shown limited utility to date. Arguably, this limitation is the result of a host of factors, the most significant of which is the lack of power that results from testing extremely rare genetic variations in case-control samples. With this in mind, family-based study designs are increasingly being considered in an effort to potentially gain a better understanding of the role that rare genetic variation plays in explaining the heritability of common disease.

Despite this renewed interest in family-based designs, few multimarker, rare-variant association tests for family-based data are available. We now briefly describe some recently proposed methods in an effort to identify methods appropriate for analyzing a categorical phenotype (hypertension) for relationships with rare variants in arbitrarily complex pedigrees (eg, multigenerational, not nuclear, family) as required for our analysis of the Genetic Analysis Workshop 18 (GAW18) data. Two recent papers [2,3] propose methods of analyzing rare variants for quantitative traits in nuclear families. However, the methods described are not applicable to binary traits or more complex pedigrees. Another recent paper proposed a method that utilizes family data to estimate SNV weights when testing case-control data, but the family data is used only to estimate weights, and case-control data is required to use the method [4]. Recently, Schifano et al [5] extended the popular SNV-set testing methods based on the kernel machine framework, used for both common and rare-variant tests of association. This paper does not reference software, so we do not consider it in this analysis.

The only paper containing methods meeting our criteria for inclusion (binary phenotypes, complex pedigrees, and available software) is by Zhu and Xiong [6], who propose a set of methods that are applicable for categorical traits in complex pedigrees. Their methods generally can be described as adjusting the standard error of the null distribution using the kinship matrix in order to account for the additional correlation observed in the sample as a result of the complex pedigree structure.

In this paper, we will apply the methods of Zhu and Xiong to investigate potential relationships between simulated and real phenotypes and genes, where genes consist of sets of SNVs within the gene. In particular, we are curious about the ability of the methods to maintain type I error and yield reasonable power for simulated phenotypes. Furthermore, through our analysis of the real phenotype, we will explore potential insights into disease etiology for hypertension using the GAW18 data.

## Methods

### Sample and genes

In the GAW18 data, real phenotype data was available for 855 individuals in pedigrees. We classified each of the 855 individuals as either ever-hypertensive or not-ever-hypertensive based on whether the individual was classified as hypertensive at any of up to 4 measurements (waves) at which phenotypic data was collected. At 849 individuals, the simulated phenotype sample was slightly smaller. When analyzing the simulated phenotype, we again focused on the (similarly defined) hypertension phenotype. In the GAW18 data, there were 200 separate phenotype simulations. Our analysis considers only simulated phenotype #1.

SNVs were mapped to genes using a custom implementation of ANNOVAR, where an SNV is assigned to a gene if it is contained within the start-stop position of the gene [7]. To improve computational time in this preliminary analysis, we restricted the analysis to 6625 genes containing between 2 and 200 SNVs.

### Statistical tests

We applied 3 different family-based tests of association considered by Zhu and Xiong [6]. The following sections briefly summarize the methods. All tests were conducted using software provided by Zhu and Xiong, which is written in R, with further data analysis conducted using our own custom R scripts. Most of the methods considered by Zhu and Xiong utilize a correction factor, *P_corr_*, which summarizes the additional correlation in the samples occurring because of the complex pedigree structure. *P_corr _*is a function of 2 times the kinship matrix. We used 2 approaches to compute the kinship matrix: (a) the theoretical kinship matrix as computed using the kinship2 R package based on stated genetic relatedness of the individuals from GAW18 documentation and (b) the estimated kinship matrix computed by estimating genetic relationships across 732,185 SNVs randomly sampled across the genome using KING [8]. We briefly describe each test below.

### The generalized T^2 ^test

Zhu and Xiong [6] propose a generalized *T^2 ^*test, which is a multivariate test comparing the mean allele counts across *t *variants (eg, SNVs within a gene) between the cases and controls; it is a multivariate version of the 2-sample *t*-test. Li and Leal [9] considered the *T^2 ^*test for case-control data. Zhu and Xiong prove that dividing the *T^2 ^*test statistic by *P_corr _*properly accounts for the pedigree structure yielding a *t *df chi-squared test statistic assuming additive polygenic inheritance.

### Combined multivariate and collapsing test for families

Of course, the downside to the *T^2 ^*test is that in the presence of rare variants there can be power loss from the large number of rare variants (*t*) present, and the asymptotic distribution of the statistic may not be valid when many extremely rare variants (eg, singletons) are present. Thus, Li and Leal [9] proposed the combined multivariate and collapsing (CMC) test whereby SNVs with minor allele frequencies (MAFs) below a particular threshold are "collapsed" into a single super variant. The generalized *T^2 ^*test is then applied to the (partially) collapsed data (the super variant plus all individual variants above the threshold). Zhu and Xiong show that, similar to the *T^2 ^*test, adjusting the CMC *T^2 ^*statistic using *P_corr _*properly accounts for the pedigree structure. In our implementation of CMC, we used 2 MAF cutoffs: 5% and 0.5%.

### $\chi_{\text{min}}^{2}$

For comparison, Zhu and Xiong also consider taking the minimum *p *value from all single-marker tests within the gene. Zhu and Xiong compute a single-marker *p *value using either a Pearson $\chi^{2}$ test or Fisher's exact test (depending on sample size) and then adjusting the *p *value using *P_corr_*. To generate a gene-based *p *value, we follow Zhu and Xiong and use the minimum *p *value across all variant sites within the gene. However, because we do not correct for multiple testing, we expect the type I error rate to be inflated using this approach.

## Results

Our analysis consisted of 2 main parts: using the simulated phenotype and using the real phenotype to investigate potential genotype-phenotype relationships in the sample. The results section is structured accordingly.

### Simulation results

We started by applying each of the tests described above to all 6625 genes, in particular, testing for association between the genotypes of the SNVs in each gene with the simulated hypertension phenotype. Table 1 shows the results of this analysis stratified by whether the gene does (171 genes) or does not (6454 genes) contain at least 1 causal SNV. Table 1 provides the percent of significant SNVs for genes containing both causal and noncausal SNV rates using a nominal α = 0.05.

**Table 1:** Percent of significant genes at α = 0.05

	6454 Genes not containing a causal SNV		171 Genes containing at least 1 causal SNV	
		
Method	Estimated kinship	Real kinship	Estimated kinship	Real kinship
$\chi_{\text{min}}^{2}$	40.6% (2621/6454)	34.4% (2221/6454)	41.5% (71/171)	33.9% (58/171)
T^2^	5.2% (333/6454)	3.2% (209/6454)	1.8% (3/171)	1.2% (2/171)
**CMC_5%_**	4.1% (267/6454)	3.3% (210/6454)	2.9% (5/171)	2.3% (4/171)
**CMC_0.5%_**	5.3% (341/6454)	3.6% (235/6454)	2.9% (5/171)	2.3% (4/171)

As expected, $\chi_{\text{min}}^{2}$ does not properly control the type I error rate, but tests using the estimated kinship provide good control of the type I error rate, and tests using the real kinship matrix (ie, the kinship matrix based on stated relationships) appear slightly conservative. Overall, the *p *values from the tests using the real and estimated kinship matrices are highly correlated (Pearson and Spearman correlations are all 0.99), with the estimated kinship matrices providing lower *p *values on average. Thus, we will use the estimated kinship matrix for all subsequent analyses. Because of the inability of $\chi_{\text{min}}^{2}$ to control the type I error rate, we do not consider that test in further analyses. Across all 6625 genes the Pearson correlation between *p *values from T^2 ^and CMC_5% _was 0.55 (Spearman = 0.54); between T^2 ^and CMC_0.5% _0.8 (Spearman = 0.80); and 0.67 between CMC_5% _and CMC_0.5% _(Spearman = 0.67).

Table 1 also illustrates that the power across the 171 genes that contain at least 1 causal SNV is essentially equivalent to the type I error rate. The low power may be attributed in part to the fact that the average variance in blood pressure explained by the causal SNVs in these genes was only 0.04% (SD = 0.1%, range: 0% to 0.9%). However, there was modest overlap in the causal genes identified as significantly associated with the phenotype. For example, when using the estimated kinship matrix, 1 causal gene, *EPHA4*, had a *p *<0.05 for all 3 approaches, which controlled the type I error rate (*T*^2^, CMC_5%_, and CMC_0.5%_). Of the remaining genes identified as significant by at least 1 of these 3 methods, most (6 of 10) had *p *values less than 0.10 for 1 of the other 2 methods, and all 10 had *p *values less than 0.18 for at least 1 of the other 2 methods.

### Real data analysis

When we applied the 3 remaining tests to the real hypertension phenotype on the entire sample of 855 individuals using the estimated kinship matrix, no minimum *p *values were below a Bonferroni-corrected alpha value of 0.05/6625 = 7.5 × 10^−6 ^for the 6625 genes. The minimum *p *value for any gene with CMC_5% _was 1 × 10^−4^. For T^2 ^there were 5 genes with *p *values less than 1 × 10^−4 ^(*MYBPHL, ZNF496, TRAT1, DHX8, ST6GALNAC2*), with 1 of these (*MYBPHL*) being the only gene for CMC_0.5%_% with a *p *value less than 1 × 10^−4^.

## Discussion

Few methods currently allow for the analysis of binary phenotypes with rare variants in complex pedigrees. We have applied 4 of the published methods to both real and simulated phenotypes. While the methods appeared to control the type I error rate for smaller genes (less than 200 SNVs), they performed poorly (low power) when the variance in blood pressure was small. Although this finding is based on the simulation model for GAW18 data, power to detect causal genes may still be a significant hurdle in real analyses.

We considered the use of both the theoretical and estimated kinship matrices. We found that use of the theoretical kinship matrix proved slightly overconservative, while using the estimated kinship matrix provided empirical type I error rates in line with the nominal levels. Even though the precise reasons for the overconservative nature of the theoretical kinship are unknown, we surmise that cryptic relatedness and population stratification between pedigrees, which are not reflected in the theoretical kinship matrix, will be controlled through use of the estimated kinship matrix. Because these issues may be present in any data set, we recommend estimating kinship matrices in practical applications of family-based tests of association for rare-variant data.

Even though minor differences in power existed across the 3 tests considered here (T^2^, CMC_5%_, and CMC_0.5%_), each test will be most powerful for particular genetic architectures. In particular, for truly causal genes the proportions of causal rare or common variants versus noncausal rare and common variants will determine which method is best. Because the "best" method here will be a product of the simulation strategy taken, we do not wish to extrapolate this simulated genetic architecture to suggest the use of one approach over the others.

A companion paper [10] demonstrates that the type I error rate for all methods considered here occurs as the number of SNVs in the gene increases beyond 200. This inflation is partly caused by the small sample size (n = 855) relative to the large number of SNVs in these causal genes. We consider alternative approaches in Ref. [10] for genes containing more than 200 SNVs.

In applying the 3 tests to the real phenotype, we found modest evidence of a relationship between a handful of genes and hypertension. These genes are featured in a modest amount of prior literature, suggesting their potential association with blood pressure, cardiovascular development, and renal function. In particular, *ZNF496 *is associated with preeclampsia (ie, hypertension during pregnancy) [11] and malignant pheochromocytoma tumors, which can lead to malignant hypertension [12]. *ST6GALNAC2 *is associated with susceptibility to IgAN, which is commonly associated with hypertension (eg, see Ref. [13]). *MYBPHL *is associated with low-density lipoprotein (LDL) cholesterol [14]. *TRAT1 *and *DHX8 *have little exposure in the literature related to hypertension or related outcomes. We note, however, that none of these genes reached a conservative (Bonferroni-corrected) significance threshold, nor did we replicate SNVs known to be associated with hypertension in large genome-wide association studies.

In addition to the lack of power from small sample size and, potentially, the choice of analysis methods, we note that our approach did not consider covariates because the methods considered in this paper do not provide obvious approaches to control covariates. As noted in the introduction, there is a dearth of methods available for the analysis of data like that in GAW18, and we know of few methods currently available that work for analysis of rare variants with binary traits on complex pedigrees, allowing for control of covariates and for which software is publicly available. With increased interest in family-based designs for the analysis of rare-variant data, further methodological development is needed in this area.

## Conclusions

The application of methods for the analysis of rare-variant data collected on complex pedigrees for relationship with a binary phenotype suggests the potential for modest power for large variant effects with a sample of 855 individuals, but minimal power for variants and genes with weaker effects. Application of the methods to a real phenotype found modest evidence of association between the hypertension phenotype and five genes, three of which have limited prior association with hypertension-related phenotypes. Further methodological work is needed to develop more powerful methods allowing for control of covariates in the analysis of complex pedigrees and for use on larger genes.

## Competing interests

The authors declare that they have no competing interests.

## Authors' contributions

All authors participated in design of the overall study; AL and AB carried out various aspects of data preprocessing, including gene mapping and building preliminary data analysis files; BG estimated the kinship matrices; CA, AH, and NT ran statistical tests, analyzed data, and conducted literature review. NT drafted the final manuscript. All authors read and approved the final manuscript.
